# The effect of testosterone on the gut microbiome in mice

**DOI:** 10.1038/s42003-024-06470-5

**Published:** 2024-07-19

**Authors:** Lelyan Moadi, Sondra Turjeman, Nofar Asulin, Omry Koren

**Affiliations:** 1 Azrieli Faculty of Medicine, Bar-Ilan University, Safed, Israel; 2 Kyung Hee University, Seoul, Republic of Korea

**Keywords:** Microbiome, Metabolomics

## Abstract

The role of hormones in gut–brain crosstalk is largely elusive, but recent research supports specific changes in hormone levels correlated with the gut microbiota. An interesting but unstudied area in microbial endocrinology is the interplay between the microbiota and sex hormones. The aim of this study is to investigate the effect of testosterone and sex on the mouse gut microbiome. We use in vitro experiments to test direct effects of testosterone on bacteria in fecal samples collected from male and female mice pre- and post-puberty. Sex-specific microbial and metabolic differences surrounding puberty are also examined in vivo. We then explore effects of testosterone supplementation in vivo, characterizing microbiota and metabolomes of male and female mice. We detect sex-specific differences in microbiota and associated metabolites of mice post-puberty, but in vitro experiments reveal that testosterone only affects microbiota of fecal samples collected before puberty. Testosterone supplementation in vivo affects gut microbiota and metabolomes in both male and female mice. Taking our results from in vitro and in vivo experiments, we conclude that the shift in the microbiome after puberty is at least partially caused by the higher levels of sex hormones, mainly testosterone, in the host.

## Abstract (web summary)

A study examining microbiome differences associated with puberty and sex and effects of testosterone on the microbiome in mice, finding shifts in the microbiome after puberty are partially caused by higher levels of sex hormones, mainly testosterone, in hosts.

## Introduction

The gut microbiota plays important roles in host metabolism, immunity, and even behavior. Mechanisms by which the microbiota mediate these functions include breaking down dietary components, priming the immune system and degrading toxins^1^. With recent technologies, large amounts of microbial data have been generated, which resulted in identification of many factors that affect the microbiome such as diet, age, antibiotic treatments, mode of delivery and more physical and environmental factors. One important factor is host sex, though there are inconsistencies between studies; only some have reported microbiota sex differences in animals and humans^2,3^.

In the Human Microbiome Project, researchers found that males were three times more likely to have lower levels of *Bacteroides* and higher *Prevotella* levels than females^2^, and a mouse study that analyzed the gut microbiome of 89 different mouse strains independently found differences in composition and diversity^3^. A critical mechanism of bacterial interaction emerged: modulation of hormonal secretion and metabolism. Surprisingly, commensal bacteria can produce and secrete hormones^4^, and the crosstalk between microbes and hormones can affect the host. This interplay is bidirectional, as the microbiota has been shown to both be affected by and affect host hormones^5^.

An interesting but understudied area in microbial endocrinology is the interplay between the gut microbiome and sex hormones. Examples of specific bacteria affected by sex hormones have been reported since the 1980s. For instance, *Prevotella intermedius* takes up estradiol and progesterone, which enhance its growth^6^. In another study, a small group of healthy women given hormonal contraceptives showed an increase in *Prevotella* species after 3 weeks of treatment, suggesting a direct impact of hormones on microbial communities^7^. We have recently shown that progesterone levels affect *Bifidobacterium* levels as well^8^, and uncovered evidence of sex-specific microbiota-mediated effects of progesterone on weight gain in mice^9^. The role of sex hormones was further illustrated in twin studies where fraternal twins with opposite sexes showed sex-biased differences in the gut microbiome after puberty compared to same sex twins^10^. Changes in expression of the estrogen receptor ER-β also affect the intestinal microbiota composition^11^. This interaction is bidirectional, as several types of bacteria have also been implicated in steroid secretion or modification. For example, *Clostridium scindens* converts glucocorticoids to androgens, a group of male steroid hormones^12^. Intestinal bacteria also play a significant role in estrogen metabolism, and antibiotic use was shown to decrease estrogen levels^13^. Furthermore, strong correlations were found between urinary estrogen levels and fecal microbiome richness, as well as presence of Clostridia and three genera within the Ruminococcaceae family^14^. One study demonstrated microbial colonization elevated testosterone levels and protected mice from type 1 diabetes and that the transfer of the male microbiome to immature females alerted their microbiota resulting in testosterone elevation and protected them from type 1 diabetes^15^.

Here, we focused mainly on testosterone, a steroid hormone from the androgen group in humans and other mammals. It is biosynthesized in several steps from cholesterol and secreted mainly by the testicles of males and, to a lesser extent, the ovaries of females. Small amounts are also secreted by the adrenal glands. It is the principal male sex hormone and an anabolic steroid^16^. Recent bioinformatic analyses identified genes that encode hydroxysteroid dehydrogenase (HSDs) in distinct bacterial genomes, some of which are members of the normal gastrointestinal microbiota^17^. Li et al. showed that gavaging rats with *Mycobacterium neoaurum* isolated from the fecal samples of testosterone-deficient patients with depression reduced their serum and brain testosterone levels and induced depression-like behaviors^18^. One study even demonstrated that *Bacillus spp*. isolated from the foregut of the water beetle *Agabus affinis* carried out 17b-reduction of androstenedione followed by D4(5)-reduction of testosterone by its functional characteristics of 14-hydroxylation, introducing ketonic function at C-6 and hydrogenation of ∆ 4-double bonds which were operative in the fermentation of both progesterone and testosterone^19^. The ability of human intestinal microorganisms to carry out reversible 17b-reduction of androgens was suggested to play a role in the regulation of testosterone levels and in the release of excess androgens in human^20^. This hypothesis was partly confirmed by the similarities of some reactions of steroid conversions by microorganisms and hormone metabolism in mammals. A recent study found that the intra-testicular levels of testosterone in germ-free mice were significantly lower than in specific pathogen-free mice^21^. Another study revealed that probiotic supplementation of *Limosilactobacillus reuteri* increased and restored testosterone levels in aging mice^22^. Interestingly, the commensal microbiota of male mice was distinct from that of females at the time of puberty, but circulating testosterone levels increased after eliminating commensal microbiota in the female mice^23^. In humans, researchers showed that gut microbiome diversity is associated with the levels of testosterone in men, and that there are significant positive correlations between serum testosterone concentrations and the relative abundance of certain genera^24^. Another study in men also showed that several bacterial families were positively associated with testosterone levels, whereas several families from the Actinobacteria, Proteobacteria, Firmicutes, and Verrucomicrobia phyla were negatively associated with testosterone levels^25^.

In this study, we examined differences in the microbiome and the metabolome between males and females and the effect of testosterone on the gut microbiome and metabolites with the ultimate goal of better understanding the role of microbiome-driven sex-biases in autoimmunity, behavior, body mass index, and disease prevalence. We hypothesized that the gut microbiota is sex dependent, and that there is a strong correlation between gut bacteria and testosterone. We thus predicted that higher levels of testosterone and dihydrotestosterone can modulate the gut bacteria composition. We found that the gut microbiome changes after puberty were mainly caused by higher levels of sex hormones and also found that direct exposure of the pre-puberty mice fecal bacteria to testosterone affected bacterial composition. We validated these findings in vivo in both male and female mice.

## Results

### Differences in the gut microbiome occurs after puberty

**Fig. 1:**
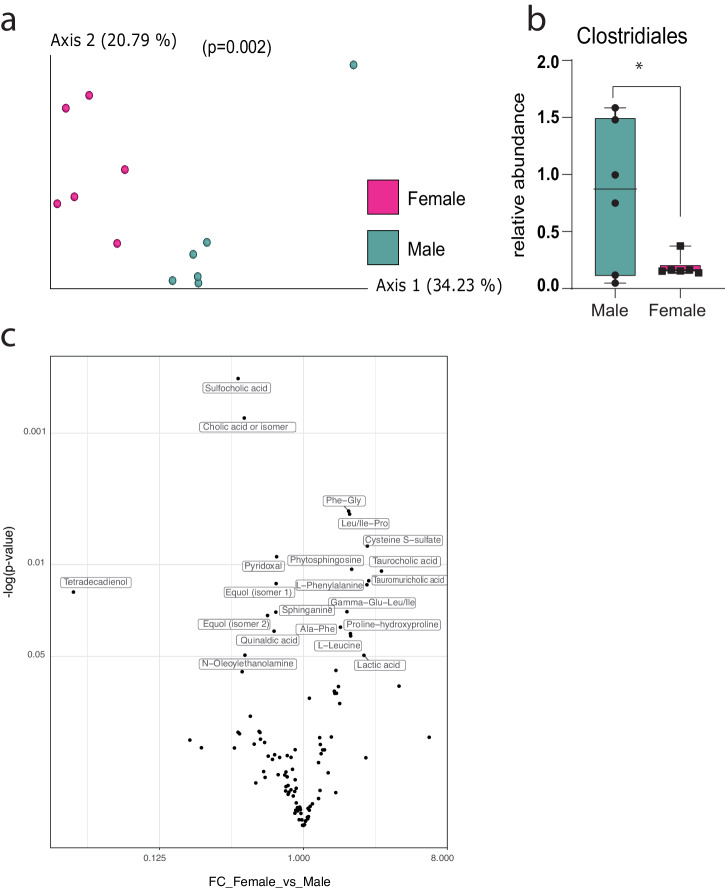
Differences in the microbiomes of male and female mice. **a** PCoA based on unweighted UniFrac distances (*p* = 0.002) for 9-week-old male and female mice (*n* = 6) and **b** the differentially abundant class, identified by ANCOM (*W* = 5). **c** Volcano plots of significantly different metabolites from the comparison between female and male mice (*n* = 4).

When comparing sex-associated changes in the gut microbiome (16S rRNA V4 gene sequencing) of male and female mice at five weeks of age, prior to puberty, no significant differences were detected in alpha- (within sample, Mann–Whitney, *p* = 0.27) or beta- (between sample, PREMANOVA, *p* = 0.09) diversity (Supplementary Fig. 1). However, when comparing samples collected from the same mice after puberty, at 9 weeks of age, although alpha diversity was still similar (*p* = 0.2), the beta diversity of the groups was significantly different (*p* = 0.002; Fig. 1a). Differential abundance analysis revealed that after puberty, male mice had higher relative abundance of the class Clostridiales than female mice (Fig. 1b). Twenty named metabolites (untargeted fecal metabolomics) were significantly different between males and females at this time-point as well: 11 were increased in females compared to males – most are essential amino acids – and 9 were increased in males compared to females – most are fatty acids, lipids, and bile acid biosynthesis metabolites (Fig. 1c).

**Fig. 2:**
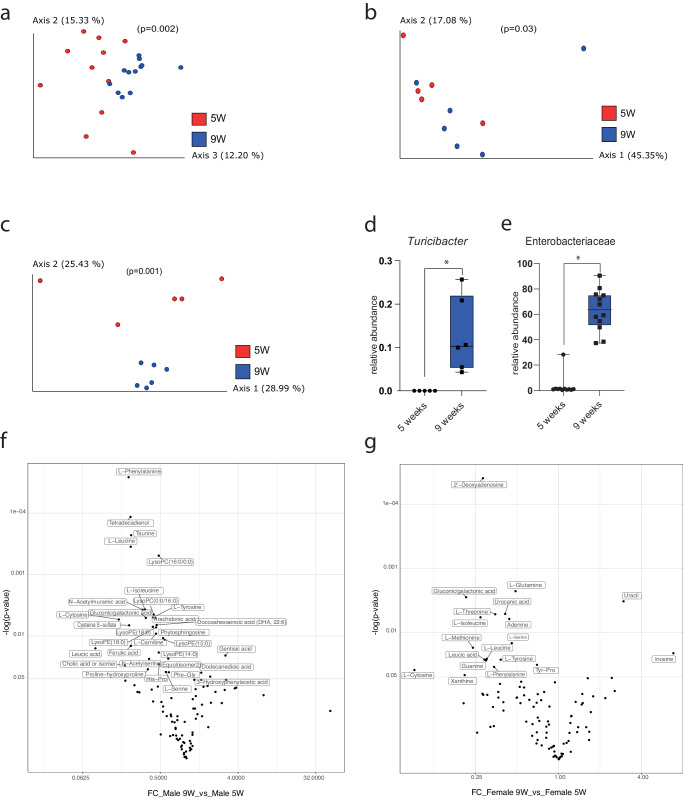
Differences in the microbiomes of 5-week-old and 9-week-old male and female mice. PCoA of unweighted UniFrac distances comparing microbiota of (**a**) all mice (*n* = 12) (*p* = 0.002), **b** male mice (*n* = 6) (*p* = 0.003), and **c** female mice (*n* = 6) (*p* = 0.001) pre- and post-puberty. Differential abundance analyses revealed relative increases in two taxa, **d** *Turicibacter* (*W* = 68) and **e** Enterobacteriaceae (*W* = 66) before and after puberty among females only. Volcano plots of significantly different metabolites from the comparison (**f**) males at 9 weeks compared to males at 5 weeks (**g**) females at 9 weeks compared to females at 5 weeks (*n* = 4).

When looking at overall changes in microbial composition pre- and post-puberty, regardless of sex, we found significant differences in beta-diversity (*p* = 0.002; Fig. 2a). This pattern was also observed when considering only males (PREMANOVA, *p* = 0.03; Fig. 2b) and only females (PREMANOVA, *p* = 0.001; Fig. 2c). In females, *Turicibacter* and Enterobacteriaceae relative abundance increased after puberty (Fig. 2d, e). No differentially abundant taxa were identified in males following puberty. The metabolomic analysis also revealed sex-specific changes in the pre- and post-puberty groups (Fig. 2f, g, respectively). A total of 32 and 19 metabolites significantly changed in the control group of males and females respectively. While a majority of amino acids and nucleotides decreased in abundance in the female group after puberty, in the male mice, lipids and amino acids and their derivatives decreased significantly.

### Testosterone supplementation changes the microbiome composition

**Fig. 3:**
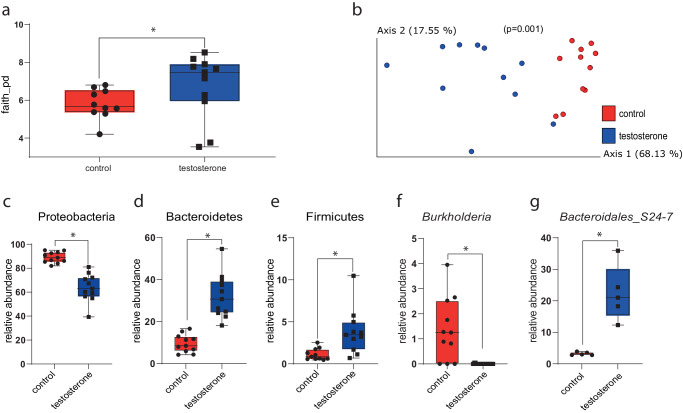
Differences in the microbiota of pre-puberty fecal slurries supplemented with testosterone compared to control samples. **a** Alpha-diversity measured using Faith’s PD (*p* = 0.032); **b** PCoA of weighted UniFrac distances of samples treated with testosterone and controls (*p* = 0.001); **c**–**e** Phylum-level relative abundance analysis using ANCOM revealed lower abundance of (**c**) Proteobacteria (*W* = 4) and higher abundance of (**d**) Bacteroidetes (*W* = 3) and (**e**) Firmicutes (*W* = 3) in males and females together (*n* = 12). **f** At the genus level, abundance of *Burkholderia* in the control group was higher compared to the treatment group in males and females together (*W* = 54), and **g** *Bacteroidales_S24-7* was higher in the treated group in females (*n* = 6) (*W* = 8).

After identifying sex-based and puberty-derived changes in mouse microbial and metabolite compositions, we examined how in vitro testosterone supplementation affected fecal microbiota in samples collected from male and female mice before and after puberty. Fecal slurries of these samples mixed with testosterone had significantly greater alpha-diversity than the control group, without testosterone (Fig. 3a). The microbiomes from the treatment group (males and females together) were significantly more similar to each other than to the control group (PREMANOVA, *p* = 0.001; Fig. 3b). When considering the sexes separately, this effect was preserved (Supplementary Fig. 2). At the phylum level, Proteobacteria relative abundance was significantly lower in the testosterone-supplemented group while Bacteroidetes and Firmicutes relative abundances were significantly higher for males and females together (Fig. 3c–e). At the genus level, the relative abundance of *Burkholderia* was significantly higher in the control group for both sexes together (Fig. 3g). In samples collected from females, the relative abundance of *Bacteroidales S24_7* was significantly higher with testosterone supplementation (Fig. 3f). Testosterone supplementation did not affect the microbiome of the samples collected after puberty in regard to alpha diversity, beta diversity, or differentially abundant taxa.

### Dihydrotestosterone supplementation changes the microbiome composition

**Fig. 4:**
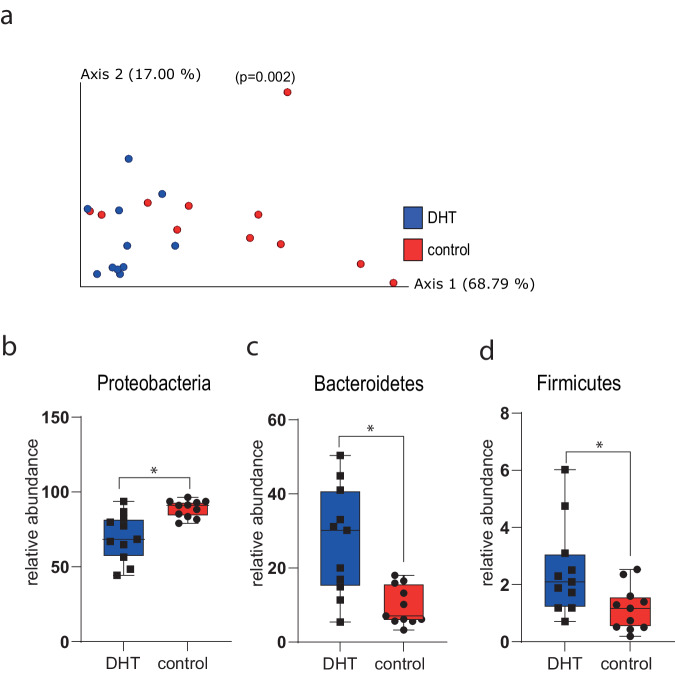
Differences in the microbiota of pre-puberty fecal samples treated with DHT and control samples. **a** PCoA of weighted UniFrac distances (*p* = 0.002); ANCOM analysis revealed significant differences in the relative abundance of (**b**) Proteobacteria (*W* = 2); **c** Bacteroidetes (*W* = 3); and **d** Firmicutes (*W* = 3) (*n* = 12).

In addition to testosterone supplementation, we also examined the effect of in vitro dihydrotestosterone (DHT) supplementation on the stool microbiota of male and female mice before puberty and after puberty. There were no significant differences in alpha-diversity, but beta-diversity of pre-puberty fecal slurries supplemented with DHT were significantly more similar to each other than to the control group (PREMANOVA, *p* = 0.002; Fig. 4a). Differential abundance tests demonstrated higher abundance of Proteobacteria in the control group (Fig. 4b) while Bacteroidetes and Firmicutes were relatively higher in the DHT-treated group (Fig. 4c, d). Like with testosterone, no significant differences were found in the microbiota of fecal slurries made from fecal samples collected after puberty and supplemented with DHT compared to the control group.

### Testosterone supplementation changes microbiome composition in mice

**Fig. 5:**
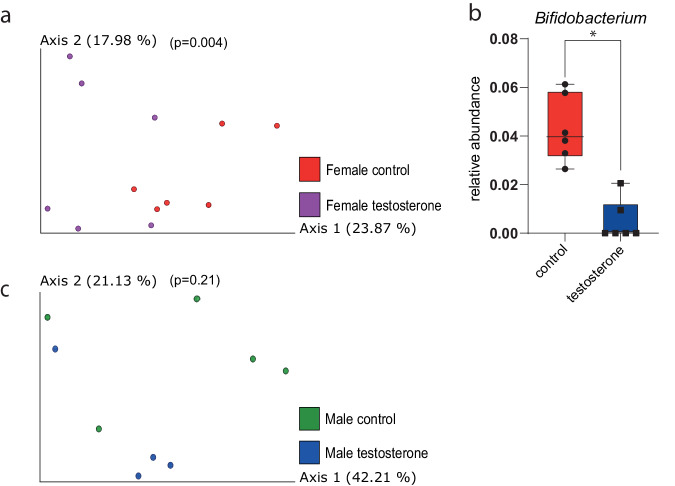
Differences in the microbiome of female and male mice 3 days after testosterone treatment. **a** PCoA of unweighted UniFrac distances of female mice (*n* = 6) treated with testosterone and controls 3 days after injection (*p* = 0.004); **b** in females *Bifidobacterium* relative abundance was significantly greater in the control group three days post-injection (*W* = 5). **c** PCoA of unweighted UniFrac distances of male mice (*n* = 6) treated with testosterone and controls three days after injection (*p* = 0.21).

**Fig. 6:**
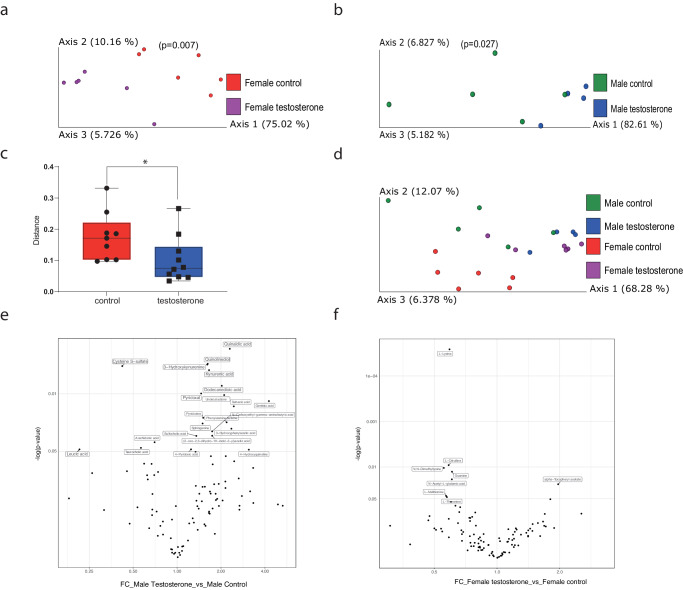
Differences in the microbiome of female and male mice after testosterone treatment. On day 21, treated female (**a**, day 21) and male (**b**, day 21) mice (*n* = 6) exhibited significantly different bacterial communities compared to controls based on weighted UniFrac (*p* = 0.007, *p* = 0.027, respectively). **c** Pairwise analysis of the weighted distance changes between paired samples (males and females together) before and after treatment showed that the microbiota of control mice changed significantly more (*p* = 0.02). **d** PCoA of weighted UniFrac distance matrices of considering sex and treatment for each treatment group on day 21. Volcano plots of significantly different metabolites from the comparison between (**e**) male control T21 and male testosterone T21 (**f**) female control T21 and female testosterone T21 (*n* = 4).

We next examined in vivo effects of testosterone supplementation on the mouse microbiome. We compared the microbiomes of male and female mice at 6 weeks, before testosterone injection and found no significant differences between the treatment and the control groups or between the sexes. Following a single injection, female mice exhibited microbial changes: the gut microbiomes of female mice 3 days after the first testosterone treatment were more diverse compared to the control group (PREMANOVA, unweighted UniFrac; *p* < 0.004; Fig. 5a). Differential abundance analysis revealed that the relative abundance of *Bifidobacterium* was higher in the control group compared to the testosterone-treated group after the first treatment (Fig. 5b). Contrarily, in male mice, there was no significant change in the microbiome after one treatment (Fig. 5c). When comparing the samples collected at later time points (days 7, 12, 15), there was initially no significant difference between the groups for both male and female mice, but on day 20/21, the beta diversity was significantly different between the treated group compared to the control group for both females (Fig. 6a) and males (Fig. 6b). Pairwise analysis that assesses the distance changed between paired samples before (day 0) and after (day 21) treatment showed that the distance between microbiome compositions was significantly greater in the control group than the treated group using (Fig. 6c, both sexes together). On day 21, the microbiomes of female mice treated with testosterone were more similar to those of treated and untreated male mice than to control female mice (Fig. 6d). Eight and 22 metabolites were significantly changed while comparing the end point measurements between the control and testosterone intervention groups in the male (Fig. 6e) and female samples (Fig. 6f). Eighteen metabolites decreased in the male testosterone group; while four metabolites (2 amino acid derivatives and 2 lipids) increased. On the other hand, six amino acids and a nucleobase were increased in the female intervention group compared to the female controls. Only, alpha-tocopherol was decreased. Interestingly, no metabolites were commonly shared between the male and female comparisons.

## Discussion

Sex differences are salient factors determining prominent features of host physiology and behavior, yet few studies have identified the effect of sex hormones on the gut microbiome. Here we examined the association between testosterone, sex differences and the microbiome through sets of in vitro and in vivo experiments. Overall, we found support for our hypothesis that testosterone affects the gut microbiome and the metabolites in a sex-specific manner.

Our results demonstrate differences in the microbiome composition and the metabolite profile between male and female mice under normal developmental conditions (no experimental interventions). The metabolite equol was increased in male fecal samples compared to females and is known to bind to DHT and to affect the prostate^26^. Interestingly, the differences in microbiome exist only after puberty, which corroborates the findings of Yurkovetskiy et al. who also showed that after puberty, gut microbiota differed in male and female mice and that there was higher microbial diversity in female mice than in males^24^. We found that male microbiomes are more similar to each other than to female microbiomes following puberty and that the relative abundance of Clostridiales is higher in males. There is accumulating evidence of sex-based differences in the microbiome, although specific findings vary^11,14,27^. This variation may be expected, though, because sexual dimorphism in the gut microbiome may result from sexual dimorphism in other environmental and physiological aspects like diet, age of puberty onset, obesity, ethnicity, and genotype. A study that tested the association of age, sex, and gut bacterial alpha diversity in three large cohorts of adults from four geographical regions found sex-dependent differences that were more pronounced in younger adults, with women having higher alpha-diversity than men^28^. Sex was also among the ten factors that most explained variability in human gut microbiota composition in a study of nearly 4000 Europeans^29^. Next, we show that the microbiome and the metabolite profile change in both males and females after puberty, and after puberty, female mice have a higher abundance of *Turicibacter* which has been linked to depression and obesity in mice^30^.

Taking together that the microbiome changes after puberty and that sex differences in the microbiome exist only after puberty, and given that samples were collected from the same mice raised under the same conditions for the whole duration of the experiment, sex hormones are the most likely factor driving the changes we detected in the microbiome. Levels of sex hormones are dramatically elevated after puberty^31^, resulting in downstream effects on the microbiome. One study found that non-pubertal subjects had higher levels of the genus *Coprobacillus* compared to pubertal subjects, and the pubertal subjects had significantly more Burkholderiales than the non-pubertal subjects^32^.

When examining the effects of testosterone in vitro, we only found effects on the microbiomes of samples collected before puberty, a time in which mice only have very low testosterone levels in the body. Samples collected after puberty were not affected by testosterone, presumably because the gut microbiota after puberty is already exposed to high levels of sex hormones in both males and females and high testosterone levels particularly in males before sample collection, thus negating any effects of added testosterone. We show that at the phylum level, testosterone treatment increases the relative abundance of Bacteroidetes and Firmicutes and decreases Proteobacteria. At the genus level, testosterone supplementation decreases the relative abundance of *Burkholderia* in males and females and increases the relative abundance of *Bacteroidales S24-7* in females. A recent study showed that the gut microbiome in women was characterized by a lower abundance of Bacteroidetes^33^, and another study showed estradiol was associated with an increased abundance of the S24-7 family, in mice^34^.

When we exposed the microbial samples to dihydrotestosterone (DHT) in vitro, our findings were in line with those from the above testosterone supplementation in vitro experiment. At the phylum level, the DHT supplementation increased the relative abundance of Bacteroidetes and Firmicutes and decreased the levels of Proteobacteria. These results indicate that testosterone and DHT have a similar effect on the microbiome. Testosterone is converted to DHT by the enzyme 5α-reductase, which is produced in many tissues in both males and females, explaining the response in both sexes^35^. A previous study showed that the levels of unconjugated DHT, but not testosterone, in feces substantially exceeded the corresponding serum values in men and mice, which suggests the ability to convert testosterone to DHT originates in the gut^36^.

In our in vivo experiment, we showed that in female mice, one injection of testosterone is enough to affect the gut microbiome, likely because their baseline levels are much lower than in males. The microbiomes of female mice treated with testosterone were more similar to each other than the untreated group, and the relative abundance of *Bifidobacterium* decreased in the treated group. It has been shown that *Bifidobacterium* is more abundant in adult females microbiome compared to males^37^, which is consistent with our results.

After 20 days of testosterone treatment, there were significant differences in the microbiomes and metabolites of the testosterone-treated group and the control group, regardless of sex. Furthermore the microbiome of female mice treated with testosterone became more similar to the male microbiome, regardless of treatment, than to the untreated female group. The metabolite cysteine-s-sulfate was significantly higher in testosterone treated mice and has been linked to increases in testosterone levels^38^, whereas in treated females, L-citrulline levels were increased compared to the controls; in the body, this metabolite is converted to L-arginine, which is associated with high testosterone levels^39^.

Most previous studies *conducted* focused on sex-specific changes in the microbiome without directly examining the effect of testosterone. One study, however, demonstrated that administration of testosterone after gonadectomy prevented the significant gonadectomy-associated changes in gut microbiota composition in specific mouse strains^3^. In line with our hypothesis that testosterone affects the gut microbiome and metabolites, the in vitro experiment performed herein offers further support that the microbiome is directly affected by testosterone. How the microbiome mediates androgen levels remains unknown, though. Future studies could include antibiotic-treated mice, germ-free mice, or expanded focuses on sex-hormone related pathways and enzymes. Despite small sample sizes, observed signals were strong, and our results are promising. We found that there are sex-associated differences in the gut microbiome after puberty and that testosterone affects the gut microbiome both in vitro and in vivo. To our knowledge, this study is the first that compared samples from the same mice to examine sex differences before and after puberty. Further research is needed to explore how the microbiome affects serum testosterone levels and to determine the significance of sex-specific differences in the microbiome on disease susceptibility and metabolic measures.

## Materials and methods

We conducted in vitro and in vivo experiments towards answering our main research goal. All animal handling and procedures described in this study were approved by the Azrieli Faculty of Medicine, Bar-Ilan University Institutional Animal Care and Use Committee (ethics protocol 20-03-2020). We have complied with all relevant ethical regulations for animal use.

### In vitro experiments

Initially, two in vitro experiments were conducted in which (1) testosterone or (2) dihydrotestosterone (DHT) was added to a solution of individual fecal samples (in PBS) collected from male and female mice before and after puberty. Fecal samples were collected from naive male and female Swiss Webster mice housed in the Azrieli Faculty of Medicine’s specific pathogen free animal facility. Samples were collected at five and nine weeks of age at the same time of the day. A portion of each sample was stored at −80 °C for later DNA extraction, to examine sex- and age-related effects on the microbiome and for metabolomic analysis (below), and the rest was used in the in vitro experiments.

We dissolved 1.5 mg of testosterone (Sigma, St. Louis, MO) in 3 ml of PBS to a final concentration of 0.5 mg/ml, and 100 µl of the solution was added to 1 ml PBS which contained an individual mouse fecal pellet (*n* = 6). Control samples were put in 1 ml of solution containing only PBS (*n* = 6). In a separate experiment, 5 µl of DHT solution (Sigma, St. Louis, MO) diluted in methanol to a final concentration of 1 mg/ml was added to fecal samples in PBS (*n* = 6) (samples from the same mice collected at the same ages as above). For the control group, 5 µl of ethanol were added to fecal samples in PBS (*n* = 6). The samples in both experiments were incubated under anaerobic conditions, at 37 °C with constant shaking for one week. Then samples were immediately processed for 16S rRNA gene sequencing (below). A separate experiment comparing effects of ethanol and methanol (vehicle) on fecal microbiota communities was also run, and no differences in community composition were identified (Supplementary Fig. 3).

### Mouse experiments: testosterone supplementation

We next examined the effects of testosterone supplementation in vivo. Six-week-old, wild-type Swiss Webster mice (*n* = 6 per treatment (testosterone injection/placebo), per sex – randomized) were housed in the animal facility under controlled temperature (22 °C) and light cycle (12 h light and 12 h dark) with free access to food (Harlan-Tekla, Madison, WI) and water. Mice were housed by group, 2 mice per cage. For each group, there were at least 3 cages to exclude any cage effects during the experiment.

Ten mg of testosterone were diluted in 2 ml olive oil to a final concentration of 0.5 µg per 100 µl; 100 µl of the solution were injected intraperitoneally to the mice every 4 days, starting from 6 weeks of age and until the mice were 9 weeks old. This is considered a high dose, given in excess with the assumption that not all of it would be absorbed into the bloodstream. The control groups were injected with 100 µl of olive oil. Fecal samples were collected 3 days after each injection and stored at −80 °C for microbial analysis. The experiment was repeated twice, and fecal samples were collected at the same intervals in both repetitions.

### DNA extraction and 16S rRNA gene amplification and sequencing

DNA was extracted from mouse stool samples collected before and after puberty and also during the testosterone supplementation study using a MagMAX Microbiome Ultra-Kit (Thermo Fisher, Waltham, MA) according to the manufacturer’s instructions and following a 2-minute bead beating step. The V4 region of the bacterial 16S rRNA gene was amplified by polymerase chain reaction (PCR) using the 515 F (AATGATACGGCGACCACCGAGATCTACACGCT) barcoded and 806 R (TATGGTAATTGTGTGYCAGCMGCCGCGGTAA) primers (10 nM)^40^. PCR reactions were carried out with the PrimeSTAR Taq polymerase (Takara, Shiga, Japan) for 35 cycles of denaturation (95 °C), annealing (55 °C) and extension (72 °C), and a final elongation at 72 °C. Products were purified using AMPure XP magnetic beads (Beckman Coulter, Indianapolis, IN) and quantified using the Picogreen dsDNA quantitation kit (Invitrogen, Carlsbad, CA). Samples were then pooled, loaded on 2% agarose E-Gel (Thermo Fisher, Waltham, MA), purified, and sequenced using the Illumina MiSeq platform (Genomic Center, Azrieli Faculty of Medicine, BIU, Israel).

### Bioinformatics analysis

Following sequencing, microbial communities were analyzed using QIIME2-2019.4^41^. Single-end sequence reads were demultiplexed, and reads errors were corrected by Divisive Amplicon Denoising Algorithm (DADA2^42^). A phylogenetic tree was generated. All analyses for mouse fecal samples were calculated based on a feature table (amplicon sequence variants, ASVs) based on the Greengenes reference database^43^ containing features observed in samples containing at least 8000 sequences; for samples from in vitro experiments, the threshold used was 7000 . All samples were rarefied to this threshold. Alpha-diversity was calculated using Faith’s phylogenetic diversity^44^ and compared using two-tailed t-tests or Mann Whitney tests, as needed. Beta-diversity was analyzed using unweighted and weighted UniFrac^45^ distances by PREMANOVA. Additionally, pairwise distance comparisons were used to assess changes between paired samples from two different time points. We also performed differential abundance testing using ANCOM^46^.

### Metabolomics analysis

#### Sample preparation

Metabolite profiles of stool samples were analyzed at Afekta Technologies Ltd. (Kupio, Finland) where samples were homogenized after adding cold 80% v/v aqueous LC-MS ultra-grade methanol in a ratio of 900 µL per 100 mg of sample for the metabolite extraction and protein precipitation using a Bead Ruptor 24 Elite homogenizer at the speed of 6 m/s at 2  ±  2 °C for 30 s. Samples were then incubated on ice for 15 min and vortexed for 10 s followed by centrifugation for 10 min at 4 °C and 17,000 × *g*. The supernatant was collected and filtered (Captiva ND filter plate, 0.2 µm) by centrifuging for 5 min at 700 × *g* at 4 °C and kept at 4 °C until analysis. Aliquots of 60 µL were taken from all samples, mixed in one tube, and prepared as reported above to be used as the quality control samples in the analysis.

#### LC–MS analysis

Samples were analyzed by liquid chromatography–mass spectrometry (LC-MS) on an Agilent 6546 Q-TOF LC/MS System with Agilent Jet Stream source and 1290 Infinity II UHPLC system. The analytical method has been described in more detail by Hanhineva et al.^47^ and Klåvus et al.^48^. In brief, a Zorbax Eclipse XDB-C18 column (2.1 × 100 mm, 1.8 µm; Agilent Technologies) was used for the reversed-phase (RP) separation and an Aqcuity UPLC BEH amide column (Waters) for the HILIC separation. After each chromatographic run, the ionization was carried out using jet stream electrospray ionization (ESI) in the positive and negative mode, yielding four data files per sample. The collision energies for the MS/MS analysis were selected as 10, 20, and 40 V, for compatibility with spectral databases.

#### Data analysis

Peak detection and alignment was performed in MS-DIAL ver. 4.90^49^. For the peak collection, *m/z* values between 50 and 1500 and all retention times were considered. The amplitude of minimum peak height was set at 3000. The peaks were detected using the linear weighted moving average algorithm. For the alignment of the peaks across samples, the retention time tolerance was 0.1 min and the *m/z* tolerance was 0.015 Da. Solvent background was removed using solvent blank samples under the condition that to be kept for further data analysis, the maximum signal abundance across the samples had to be at least five times that of the average in the solvent blank samples.

After the peak picking, a total of 62,665 molecular features were included in the data preprocessing and clean-up step. Low-quality features were flagged and discarded from statistical analyses. Molecular features were only kept if they met all the following quality metrics: low number of missing values (present in more than 70% of the QC samples, present in at least 50% of samples in at least one study group), RSD* below 20%, D-ratio* below 10%. In addition, if either RSD* or D-ratio* was above the threshold, the features were kept if their classic RSD, RSD* and basic D-ratio were all below 10%. The signals were normalized for signal drift. Missing values were imputed using simple imputation with value of 0 for all features.

After the preprocessing and data clean-up, 42,284 molecular features were considered of high quality and included in the FDR correction calculations (below). The high number of molecular features before data clean-up is due to the high sensitivity of the instrument, collecting several signals from each actual metabolite, but also from the solvent background and detector noise.

The main statistical analyses are feature-wise two-sided Welch’s t-tests run separately between two time points and between all pairs of group levels. FDR-corrected *p*-values were calculated. Fold changes were also computed for these comparisons. In addition, feature-wise linear models with testosterone, time and their interaction term as predictors of feature levels were fitted for both male and female samples separately. All statistical analyses were conducted with R version 4.1.2. All signals with raw *p*-value < 0.05 were selected for annotation.

#### Compound identification

The chromatographic and mass spectrometric characteristics (retention time, exact mass, and MS/MS spectra) of the significantly differential molecular features were compared with entries in an in-house standard library and publicly available databases, such as PubChem and HMDB. The annotation of each metabolite and the level of identification were given based on the recommendations published by the Chemical Analysis Working Group (CAWG) Metabolomics Standards Initiative (MSI)^50^.

### Supplementary information

Supplementary Information

Description of Additional Supplementary Files

Supplementary Data 1

Supplementary Data 2

Supplementary Data 3

Supplementary Data 4

Supplementary Data 5

Supplementary Data 6

Supplementary Data 7

## Data Availability

Data has been uploaded to EBI (project number ERP157003) and are available at the following URL: https://www.ebi.ac.uk/ena/browser/view/PRJEB72219. The source data behind the graphs in the paper can be found in Supplementary Data 1–7.
