# Supplementary material for: The effect of testosterone on the gut microbiome in mice
Source: Commun Biol. 2024 Jul 19;7:880. doi: 10.1038/s42003-024-06470-5 (PMC11271554; doi:10.1038/s42003-024-06470-5)
Supplement: Supplementary file 1 — Supplementary Information [file 42003_2024_6470_MOESM1_ESM.pdf]

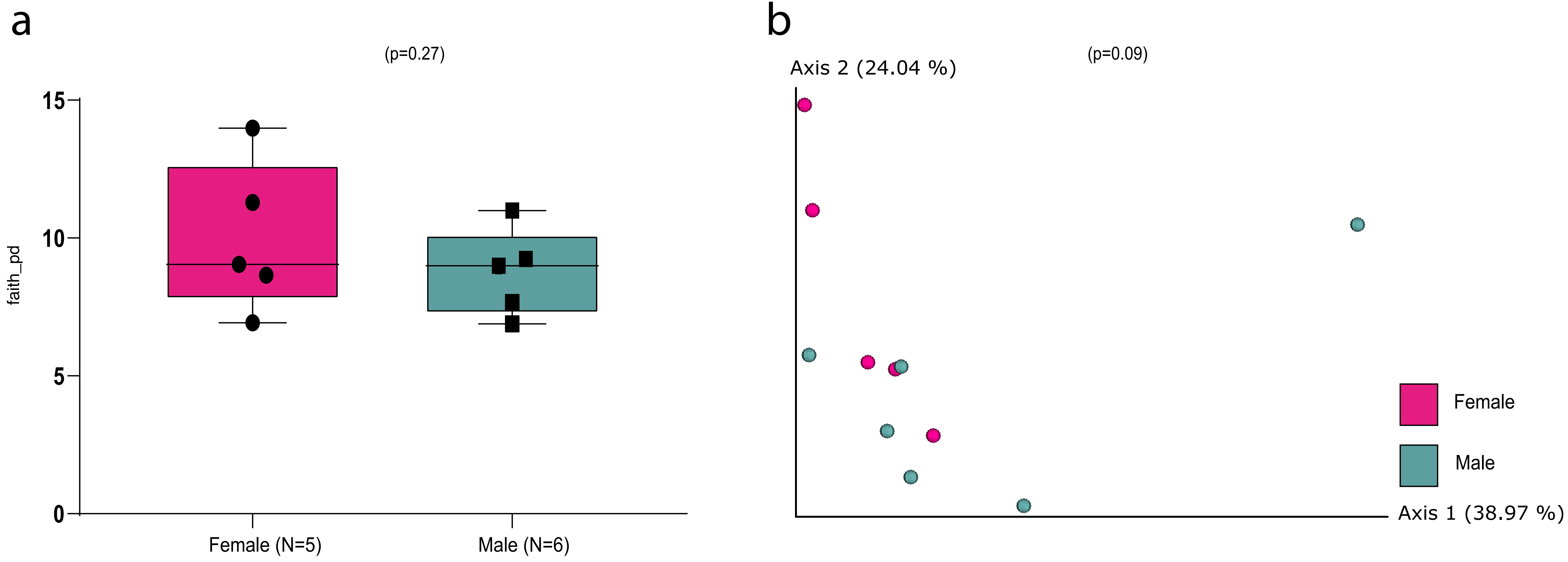

Supplementary Figure 1. The microbiomes of male and female mice prior to puberty. (a) Alpha diversity measured using Faith's PD (p=0.27) (b) PCoA based on unweighted UniFrac distance matrices (p=0.09) for 5-week-old male and female mice (n=6).

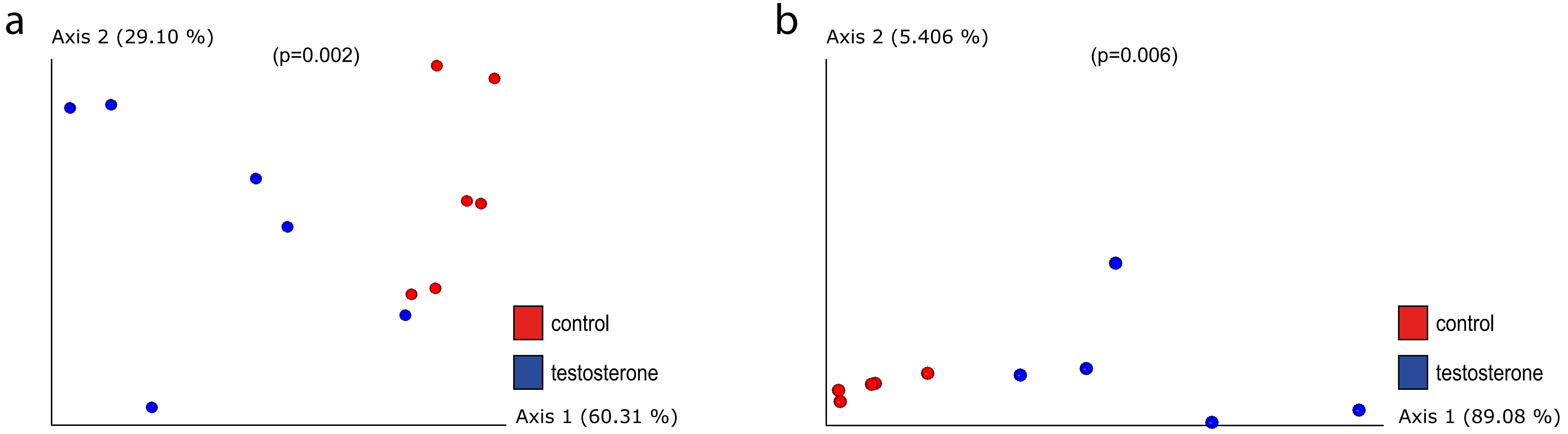

Supplementary Figure 2. Differences in the microbiota of pre-puberty fecal slurries supplemented with testosterone compared to control samples. (a) PCoA of weighted UniFrac distances of male mice samples treated with testosterone and controls (p=0.002). (b) PCoA of weighted UniFrac distances of female mice samples treated with testosterone and controls (n=6) (p=0.006).

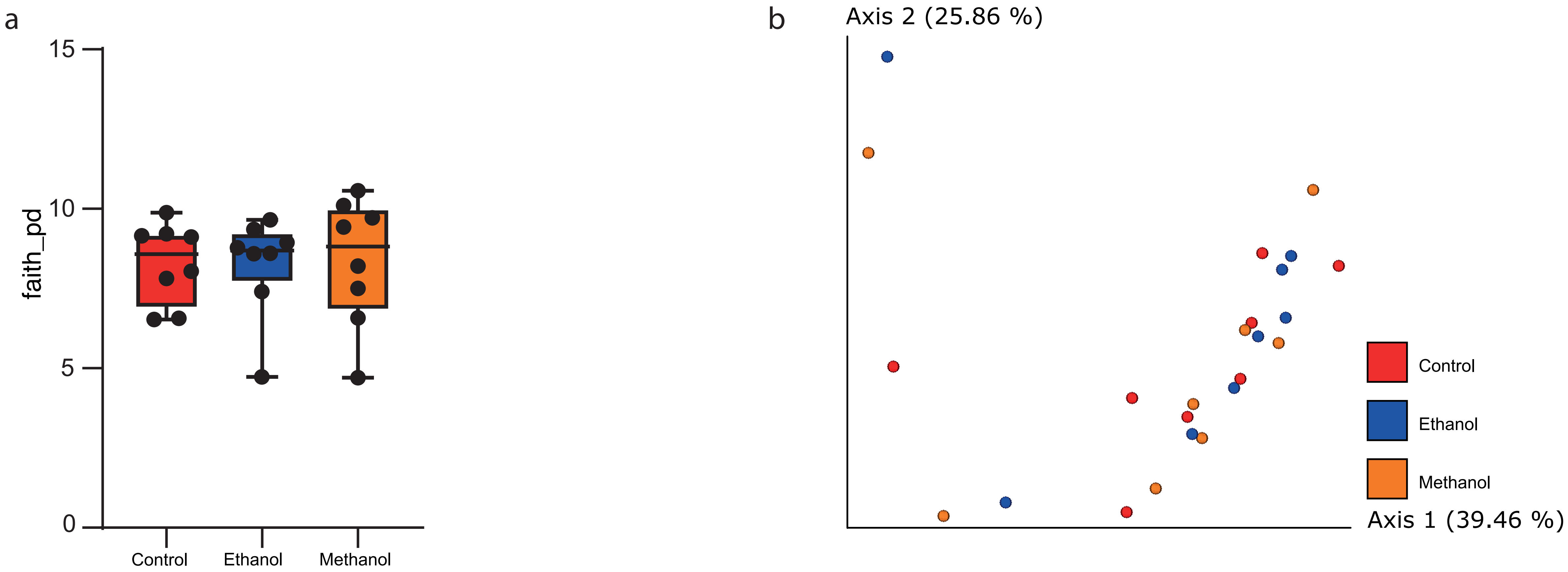

Group 1	Group 2	p-value	q-value
Control (n=8)	Ethanol (n=8)	1.000000	1.0
	Methanol (n=8)	0.528612	1.0
Ethanol (n=8)	Methanol (n=8)	0.674424	1.0

Group 1	Group 2	p-value	q-value
Control	Ethanol	0.995	0.998
	Methanol	0.978	0.998
Ethanol	Methanol	0.998	0.998

Supplementary Figure 3. Ethanol and methanol addition do not affect the bacteria composition. An in vitro experiment was conducted testing the effect of the two different vehicles used in this study, ethanol and methanol, on the bacterial community composition. The experiment timeline was like the other in vitro experiments. Three fecal samples (each) were collected from 8 experimental mice, and then diluted in 1 ml PBS. We then added 5 µl of ethanol to one sample per mouse, 5 µl of methanol to one sample be mouse, and the third sample was left with only PBS as a control. The samples were incubated for 7 days, and the DNA extraction, sequencing, and analysis were done as explained in the article (n=8). (a) Alpha diversity measured using Faith's PD (b) PCoA based on weighted UniFrac distance matrices.
