# Supplementary material for: The effect of testosterone on the gut microbiome in mice
Source: Commun Biol. 2024 Jul 19;7:880. doi: 10.1038/s42003-024-06470-5 (PMC11271554; doi:10.1038/s42003-024-06470-5)
Supplement: Supplementary file 2 — Description of Additional Supplementary Files [file 42003_2024_6470_MOESM2_ESM.pdf]

Description of Additional Supplementary Files

File name: Supplementary Data 1

Description: The source data behind Figure 1 in the paper

File name: Supplementary Data 2

Description: The source data behind Figure 2 in the paper

File name: Supplementary Data 3

Description: The source data behind Figure 3 in the paper

File name: Supplementary Data 4

Description: The source data behind Figure 4 in the paper

File name: Supplementary Data 5

Description: The source data behind Figure 5 in the paper

File name: Supplementary Data 6

Description: The source data behind Figure 6 in the paper

File name: Supplementary Data 7

Description: The source data behind the Supplementary figures
